# Nuclear Localization of Heme Oxygenase-1 in Pathophysiological Conditions: Does It Explain the Dual Role in Cancer?

**DOI:** 10.3390/antiox10010087

**Published:** 2021-01-11

**Authors:** Marilina Mascaró, Eliana N. Alonso, Exequiel G. Alonso, Ezequiel Lacunza, Alejandro C. Curino, María Marta Facchinetti

**Affiliations:** 1 Laboratorio de Biología del Cáncer, Instituto de Investigaciones Bioquímicas de Bahía Blanca (INIBIBB), Dpto. de Biología, Bioquímica y Farmacia (UNS), Universidad Nacional del Sur (UNS)-CONICET, Bahía Blanca 8000, Argentina; mmascaro@inibibb-conicet.gov.ar (M.M.); ealonso@criba.edu.ar (E.N.A.); egalonso@inibibb-conicet.gob.ar (E.G.A.); 2 Centro de Investigaciones Inmunológicas Básicas y Aplicadas (CINIBA), Facultad de Ciencias Médicas, Universidad Nacional de La Plata, La Plata CP1900, Argentina; elacunza@med.unlp.edu.ar

**Keywords:** heme oxygenase-1, nucleus, cancer, oxidative stress, nuclear protein, nuclear localization

## Abstract

Heme Oxygenase-1 (HO-1) is a type II detoxifying enzyme that catalyzes the rate-limiting step in heme degradation leading to the formation of equimolar quantities of carbon monoxide (CO), free iron and biliverdin. HO-1 was originally shown to localize at the smooth endoplasmic reticulum membrane (sER), although increasing evidence demonstrates that the protein translocates to other subcellular compartments including the nucleus. The nuclear translocation occurs after proteolytic cleavage by proteases including signal peptide peptidase and some cysteine proteases. In addition, nuclear translocation has been demonstrated to be involved in several cellular processes leading to cancer progression, including induction of resistance to therapy and enhanced metastatic activity. In this review, we focus on nuclear HO-1 implication in pathophysiological conditions with special emphasis on malignant processes. We provide a brief background on the current understanding of the mechanisms underlying how HO-1 leaves the sER membrane and migrates to the nucleus, the circumstances under which it does so and, maybe the most important and unknown aspect, what the function of HO-1 in the nucleus is.

## 1. Introduction/Heme Oxygenase-1

Heme Oxygenase-1 (HO-1) catalyzes the rate-limiting step for the degradation of heme, a potent pro-oxidant and pro-inflammatory agent, yielding equimolar quantities of biliverdin, carbon monoxide (CO) and free iron, thus acting as an indirect antioxidant defense system [1,2].

Heme Oxygenase-1 is one of the three mammalian HO isoforms identified so far. In humans, it is derived from the full-length mRNA transcript of HMOX1 gene, which is localized on chromosome 22q13.3 containing five exons and four introns, and encodes a 32 kDa molecular weight protein composed of 288 aminoacids, also known as the Heat Shock Protein 32 (HSP32) [2,3]. Of note, HO-1 is induced by different stimuli such as cellular stress, hypoxia and oxidative injury, among others. The other HO isoforms are HO-2 and HO-3. HO-2 is a constitutively expressed 36 kDa protein found in humans, rat and mouse [2,3], while HO-3, is a HO-2 derived pseudogene found in rat [4].

Heme Oxygenase-1 can be regulated at many levels. At the transcriptional level, several Transcription Factors (TFs) are able to bind to the enhancers located in HMOX1 gene in order to regulate the mRNA expression of HO-1: a Proximal Promoter (PP) at half a kilobase, Distal Enhancer E1 (DE1) at 4 kilobases, and Distal Enhancer E2 (DE2) at 10 kilobases upstream from the transcription start site. Among the TFs, AP-1 is able to bind to the three main enhancer clusters, Nrf2 binds to DE1 and DE2, and NF-kB binds to the PP [5]. These TFs belong to the antioxidant transcription factor network and are activated, depending on Reactive Oxygen Species (ROS) levels, to mitigate oxidative cell damage [6]. At the posttranscriptional level, it has recently been reported the expression of a 14 kDa HO-1 protein, generated by exon 3 skipping of HMOX1 gene, in human normal and tumor cell lines [7]. At the posttranslational level, HO-1 protein sequence shows susceptibility to be palmitoylated, acetylated, phosphorylated, ubiquitinated and truncated [8].

Since its initial discovery in 1968, HO-1 has been characterized as an Endoplasmic Reticulum (ER)-associated protein due to the abundant detection of HO activity in the microsomal fractions [1]. Later, HO-1 was found to be localized in other subcellular compartments besides the ER, including the mitochondria, the vacuole, the nucleus and the plasma membrane [8]. Interestingly, HO-1 migration from the sER to the mitochondria, the vacuole and the plasma membrane is associated with retention of its enzymatic activity whereas its migration to the nucleus is associated with loss of this function [8,9]. Currently, it is known that some modifications at the posttranscriptional and posttranslational levels are related to the subcellular distribution. For example, it has been demonstrated that a 14 kDa HO-1 form can mainly be induced by UV irradiation and by H_2_O_2_ being retained in the cytoplasm [7], whereas a 32 kDa HO-1 can be cleaved from the sER membrane, leading to a 28 KDa truncated HO-1 form, which is the main form found in the nucleus, as we describe later [10].

In this work, we provide a brief background of the current knowledge about nuclear HO-1 significance, with special emphasis on malignant processes, but also taking into account what is known about nuclear HO-1 in some physiological and other pathological conditions. In addition, we review the mechanisms underlying the migration of HO-1 from the sER membrane to the nucleus and the circumstances under which it does so, further discussing the evidence obtained so far of the biological consequences of HO-1 translocation into the nucleus.

## 2. Nuclear HO-1 Is a Truncated form with Non-Canonical Functions

Full enzymatically active HO-1 resides in the sER in an oligomeric state, where it is anchored through a single Transmembrane Segment (TMS) located at its C-terminal domain, being crucial the integrity of the sER membrane to preserve its enzymatic activity. Moreover, currently, it is known that at least the Trp-270 residue in the alfa-helix TMS is highly relevant to proper HO-1 oligomerization, enzymatic activity and its proteolytic cleavage [11,12]. As early as 1991, using mild trypsinization Yoshida et al. demonstrated that HO-1 from rat liver microsomes is sensitive to proteolytic cleavage and that a 28 KDa peptide is obtained [13]. Later, in vitro, it was demonstrated that after three different stimuli such as hypoxia exposure and treatment with heme/hemopexin and hemin, HO-1 is overexpressed and cleaved from the sER, generating a 28 kDa C-terminal truncated HO-1 (t-HO-1) form. C-terminal-truncation of HO-1 abolishes oligomerization and reduces its enzymatic activity, compared to native HO-1 [12]. Proteolytic cleavage of HO-1 can be prevented by E64d inhibitor, which inhibits cathepsin B and calpain-1 and -2, suggesting an involvement of these enzymes in releasing t-HO-1 from the sER [10,14]. Moreover, HO-1 can also be cleaved by Signal Peptide Peptidase (SPP), which associates with TCR8, an ER-resident ubiquitin E3 ligase, leading to HO-1 dislocation, ubiquitination and subsequent proteasome-mediated degradation [15,16,17]. The ubiquitin-proteasome system may be activated to degrade misfolded or damaged proteins but also to regulate physiological protein turnover in the ER, as it could occur in an HO-1 overexpressed condition in order to restore HO-1 levels [15,18]. Indeed, a PEST domain for rapid turnover of HO-1 protein has been reported [19]. Once t-HO-1 is released from the sER, it is able to translocate to the nucleus where it plays non-canonical functions.

**Figure 1:**
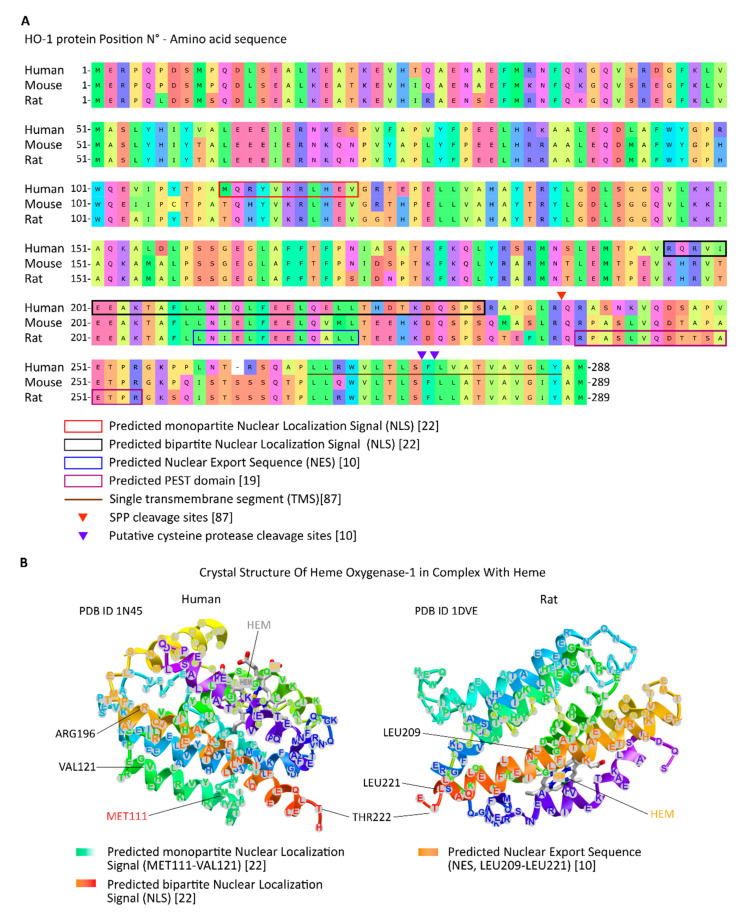
(**A**) Critical regions for proteolytic cleavage by SPP and cysteine proteases, and for proteasome degradation, as well as the TMS and the predicted nucleocytoplasmic shuttling (NES and NLS) sequences are indicated in the amino acid sequence for HO-1 protein. (**B**) Regions for predicted nucleocytoplasmic shuttling (NES and NLS) sequences and the heme-binding site are indicated in 3D models of HO-1 protein.

Protein migration to the nucleus can occur by diffusion if a protein has a molecular weight below 40 kDa or by active transport if it has a Nuclear Localization Sequence (NLS), which can bind to importin-α/β heterodimer and then go through a Nuclear Pore Complex (NPC) using the Ran system. On the contrary, a protein can translocate from the nucleus to the cytoplasm by active transport if the protein expresses a Nuclear Export Sequence (NES) that allows the protein to bind to CRM-1, also known as exportin-1, and its passage through the NPC also using the Ran system [20,21]. To date, a predicted monopartite NLS at position 111 and a predicted bipartite NLS at position 196 have been reported for HO-1 by bioinformatic analysis [22], but whether there is an importin-related mechanism implicated in nuclear HO-1 import remains to be confirmed. On the other hand, a lysine-rich region highly homologous to a NES motif on HO-1 protein has been identified and its functionality demonstrated by its interaction with CRM-1, as well as its participation in HO-1 shuttling through the nucleus [10]. Critical regions for proteolytic C-terminal truncation, protein degradation by proteasome and the nucleocytoplasmic shuttling are shown in Figure 1.

Related to its nuclear role, HO-1 protein is able to modulate TF activities. It has been demonstrated that, independently of its enzymatic activity, HO-1 protein decreases the DNA binding activity of NF-kB but increases the activation of CBF, Brn-3 and AP-1 TFs [10]. Moreover, an increase in the phosphorylation of c-Jun, a subunit of AP-1, by HO-1 has also been reported [10]. Interestingly, based on the kind of stimulus, t-HO-1 is able to protect or induce cell death. For example, under an oxidative condition such as H_2_O_2_ treatment, both full-length HO-1 and t-HO-1 expression prevent cell death. On the contrary, after hemin treatment, full-length HO-1 also protects cells from death whereas t-HO-1 increases it. This suggests that t-HO-1 has a pivotal role depending on the nature of the stimulus and possibly involving different downstream signaling pathways [10].

In another oxidant condition such as hyperoxia, the truncation of HO-1 is also induced. Indeed, it has been demonstrated that, as opposed to hypoxia, t-HO-1 induces Nrf2 expression, binds to it, and the complex migrates to the nucleus. Furthermore, t-HO-1 contributes to stabilizing Nrf2 into the nuclear compartment, preventing its proteasomal degradation mediated by PI3K/Akt/GSK3β. In addition, t-HO-1 participates in Nrf2-mediated antioxidant defense by inducing mRNA expression of G6PDH and NQO1 but no SOD2 genes. Moreover, G6PDH activity is also enhanced [23]. G6PDH is the rate-limiting enzyme in the Pentose Phosphate Pathway (PPP), a metabolic pathway through which nucleic acid precursors as well as NAPDH are synthetized. In addition, NAPDH is relevant in the maintenance of antioxidant defenses [24]. In sum, nuclear HO-1 may mediate oxidative stress protection through the transcriptional regulation of antioxidant genes instead of by means of its enzymatic activity. A similar finding was previously reported by Collinson et al. using a simple eukaryotic model as yeast [25]. Interestingly, these authors also reported that, at least in that model, HO-1 modulated a set of genes involved in RNA processing, ribosome biogenesis and transcriptional regulation, all processes associated with a nuclear location, but a small fraction of genes related with antioxidant activity [25].

In addition, full-length HO-1 and, although to a lesser extent, its truncated form, are able to activate its own promoter, which has several antioxidant responsive elements, demonstrating that HO-1 is also able to transcriptionally regulate its own expression independently of its enzymatic activity. Such activation is regulated via both the distal enhancer E1 and E2 regulatory regions and is independent of MAPK pathways, which is usually activated in response to oxidative stress [26].

The modulation of TF activity as well as the activation of its own promoter have shed some light about the nuclear role of HO-1. However, the exact molecular mechanisms by which it does so remain unknown. It is highly recognized that HO-1 protein does not have a DNA-binding consensus sequence [10]. One possibility would be that HO-1 may act as a transcriptional co-factor or may integrate a transcriptional protein complex. Moreover, the composition of such protein complex may vary depending on the initial stimuli or cell type. A nuclear HO-1 interactome would provide more accurate information about the interaction of nuclear HO-1 with other nuclear proteins.

## 3. Nuclear HO-1 in Physiological and Non-Malignant Pathological Conditions

Heme Oxygenase-1 translocation from the cytoplasmic to the nuclear compartment has been demonstrated in physiological processes such as cell differentiation in enterocytes [27], brown adipocytes [28] and astrocytes [29]. In addition, it has been implicated in some stressful events such as thermogenic stimuli in rat brown adipocytes [28], excitotoxicity in rat astrocytes [29] and, under hyperoxia, in rat and mouse fetal lungs [30,31].

In the small intestine, nuclear HO-1 expression remains low in newly differentiated cells, but such expression increases in fully differentiated and senescent cells [27]. Accordingly, our group reported nuclear HO-1 expression in human Colorectal Cancer (CRC) and in a carcinogenic murine model of CRC [32], as discussed in the following chapter. However, to date, although HO-1 was implicated in several inflammatory conditions of the intestinal tract [33,34,35,36], its subcellular compartmentalization has not been reported in such conditions.

Rat mature brown adipocytes also express nuclear HO-1 and it has been demonstrated that there is an increase in HO-1 protein and mRNA levels after a non-shivering stimulus. On the contrary, only cytoplasmic HO-1 expression was induced by noradrenergic stimulus [28]. These authors also demonstrated that HO-1 and UCP1, a mammalian thermogenic mitochondrial protein, share a staining pattern in brown adipocyte tissue after a thermogenic stimulus, suggesting that HO-1 may play a role as a protective mechanism [37]. In accordance with these results, genetic and pharmacological overexpression of HO-1 have been implicated in a better metabolic function of adipose tissue, a reduction of adiposity and an increase of insulin sensitivity impacting on diabetes, obesity and cardiovascular performance [38,39]. However, to date, the precise nuclear role of HO-1 on those processes remains to be elucidated.

In addition, in the rat cerebral cortex, nuclear HO-1 expression has been implicated in astrocyte differentiation. Indeed, HO-1 overexpression under excitotoxic conditions due to glutamate stimulus through its AMPA/KA receptors suggested an involvement of nuclear HO-1 not only in neuroglial development but also in neurodegeneration [29]. Recently, it has been demonstrated that overexpression of nuclear HO-1 promoted functional recovery of spinal cord injury by inhibiting ER-stress and apoptosis [40]. However, there needs to be a more comprehensive understanding of nuclear HO-1 in developmental and degenerative processes of the central nervous system.

In immature lungs exposed to hyperoxia, nuclear HO-1 has also been reported, and this localization favored the development of bronchopulmonary dysplasia. Rat fetal lungs exposed to hyperoxia overexpressed HO-1, which exerted a protective role. Interestingly, after 3 days of hyperoxia, HO-1 translocated into the nucleus and such migration was associated with lack of the HO-1 enzymatic activity and with oxidative injury markers returning to control values, suggesting a potential regulatory role of the nuclear HO-1 in HO-1 overexpression and consequently in its protective effects [30]. After hyperoxia-induced damage, a recovery period is necessary to re-establish lung function as shown using a mouse model of postnatal lung repair. The authors demonstrated that when HO-1 was deleted and lung tissue exposed to hyperoxia, cell proliferation and DNA damage response genes were dysregulated, thus impairing tissue repair. In this model, and in accordance with that previously observed in rat fetal lungs, nuclear HO-1 lacked enzymatic activity after hyperoxic exposure. Moreover, it has been shown that when HO-1 translocated into the nucleus, it bound to and retained hnRNPK [31]. hnRNPK is a nucleic acid-binding protein with transcriptional and translational functions, among others [41]. The nuclear retention of hnRNPK by HO-1 altered hnRNPK subcellular distribution and impaired β-catenin/hnRNPK signaling, which is relevant to lung tissue repair. This alteration in the subcellular distribution of hnRNPK contributes to explain how HO-1 led to a dysregulation of the expression of genes involved in cell cycle and DNA-damage response, thus impairing these processes needed to recover the lung tissue function [30]. Later on, it has been also demonstrated that the recovery of lung tissue depended on both the level of HO-1 expression and its subcellular location. Using the human surfactant protein-C as lineage specific marker, these authors engineered transgenic mouse lines to drive hemagglutinin-tagged full-length HO-1 and C-terminal 53 amino acid truncated HO-1 cDNAs into murine lung tissue. Interestingly, three independent mouse models were generated: one overexpressing low cytoplasmic full-length HO-1, one overexpressing high cytoplasmic full-length HO-1 and another overexpressing nuclear truncated HO-1. Low cytoplasmic HO-1-expressing mice recovered better than those expressing high cytoplasmic or nuclear HO-1. Moreover, nuclear HO-1 mice showed high DNA damage and high PARP levels but failed to hydrolyze PAR proteins, maybe due to HO-1 binding to PARG, thus impairing its enzymatic function [42].

## 4. Nuclear HO-1 in Cancer

The relevance of HO-1 in cancer has been demonstrated in several types of tumors. HO-1 may play a protumor role in most tumor types, as demonstrated in some gastrointestinal cancers [43,44,45,46,47], glioma [48], head and neck cancer [49], lung cancer [50,51], thyroid cancer [52], genitourinary cancers [53,54,55,56], melanoma [57] and hematological malignancies [58,59]. Contrariwise, an antitumor role for HO-1 has also been reported in some of the above-mentioned malignancies, such as colon cancer [32,60], hepatic cancer [61], prostate cancer [62], head and neck cancer [63] and lung cancer [64]. Interestingly, in some types of cancers, both cytoplasmic and nuclear HO-1 expressions have been reported in tumor cells. Furthermore, when HO-1 translocated from the cytoplasm to the nucleus, an alteration in cancer cell behavior was observed. This suggests that HO-1 would have a dual role in cancer that seems to depend on the tumor type, HO-1 subcellular localization, HO-1 threshold levels or a combination of them [65,66]. To date, most studies reporting nuclear HO-1 in human cancer, have evaluated the association of this localization with clinic pathological data such as tumor grade, patient survival time or differentiation grade. The knowledge of nuclear HO-1 localization significance in human tumors may lead to its potential utility as a tumor biomarker but also may shed light on the role of this HO-1 form in tumor biology. Since the role of HO-1 in cancer has been extensively reviewed, in this work, we focus on the literature where nuclear HO-1 was reported. We summarize nuclear HO-1 studies in cancer in Table 1.

The first report showing nuclear HO-1 in a tumor disease was made by Vazquez et al. which demonstrated HO-1 nuclear staining in prostate cancer [67]. Cytoplasmic HO-1 expression in human prostate cancer biopsies showed similar levels throughout tumor progression from non-tumor parenchyma and Benign Prostatic Hyperplasia (BPH) to tumor disease. However, nuclear HO-1 staining was stronger in the tumors when compared to non-malignant tissues and BPH, suggesting a role in tumor transformation. Other authors have reported a correlation between high nuclear HO-1 expression with poorer overall survival [68]. In addition, Vazquez et al. demonstrated that in vitro pharmacological treatment with hemin of androgen-sensitive (LNCaP) and androgen-insensitive (PC3) prostate cancer cell lines induced HO-1 overexpression and its nuclear translocation in both tumor subtypes [67]. Moreover, hemin-induced HO-1 expression reduced PCa cell proliferation, cell migration and invasion processes as well as pro-angiogenic genes expression. In accordance with the latter finding, HO-1 overexpression repressed the transcriptional activity of NF-kB, a TF involved in inflammation and angiogenesis. In addition, hemin treatment decreased in vivo neovascularization and tumor growth of HO-1-overexpressing PCa xenograft model. Notably, nuclear HO-1 was observed in these tumor xenografts. In this context, the expression and activity of MMP9, a downstream target of NF-kB and a well-known player in PCa spread, was also downregulated. All these results demonstrate that HO-1 plays an antitumor role in PCa [69,70]. Related to the nuclear HO-1 role, the same authors demonstrated that in testosterone-stimulated LNCaP cells, HO-1 associated with the proximal region promoter of MMP9, thus modulating its gene expression, as well as to uPA and PSA gene promoters [71]. Moreover, they showed that HO-1 bind to STAT3 retaining it into the cytoplasm, thus impairing its binding to the androgen receptor and STAT3/AR nuclear translocation, thus leading to a reduced induction of STAT3 target genes [71]. In addition, Dennery et al. demonstrated constitutive nuclear expression of a truncated (28 kDa) form of HO-1 in LNCaP prostate cancer cell line [23]. The authors showed evidence that the nuclear 28 kDa HO-1 co-immunoprecipitates with Nrf2, although this Nrf2 is not phosphorylated at Ser^40^, which is a posttranslational modification that modulates Nrf2 activity. Instead, the authors demonstrated that nuclear HO-1 stabilize Nrf2, thus regulating the transcription of specific downstream antioxidants and metabolic genes [23]. With regard to how HO-1 leaves the sER membrane in PCa, cathepsin B expression in human PCa tissues was reported [72]. However, no significant differences on calpain-1 and -2 expression in tumor versus normal samples were found [73]. Whether some of these enzymes as well as SPP are involved in the HO-1 truncation in these tumor cells remains to be demonstrated. Interestingly, in a proteomic profiling of HO-1-interacting proteins from HO-1-overexpressed PC3 cells, an enrichment in the proteins associated with DNA- and chromatin-related processes and with RNA metabolism was reported [74]. Nonetheless, the role of HO-1, and particularly t-HO-1, in such nuclear cellular processes should be thoroughly studied. To elucidate the molecular mechanism of nuclear HO-1 in PCa, Birrane et al. evaluated the effect of smoking medium (SM), which increases the risk for prostate cancer, on nuclear HO-1 translocation and VEGF secretion. They demonstrated that SM induced nuclear HO-1, which mediated VEGF secretion, thus contributing to angiogenesis. However, it is interesting to note that the authors have used a DNA construct to overexpress HO-1 with tandem nuclear localization sequence at the C-terminus. We wonder if, in this case, it is possible that nuclear HO-1 keep the native enzymatic activity and the observed effects be mediated by HO-1 by-products instead of by truncated HO-1 per se [75].

**Table 1:** Evidence showing nuclear HO-1 expression in cancer.

Tumor Type	Tissue Compartment Expressing Nuclear HO-1	Proposed Role for Total or Cytoplasmic HO-1	Proposed Nuclear HO-1 Role	Evaluation of Nuclear HO-1 Expression and Clinic Pathological Parameters	References
Lung SCC	Tumor tissue	Protumor	Protumor	Tumor stages I, II and III (versus non/malignant tissue) **	[76]
NSCLC	Tumor tissue and non-malignant tumor tissue	Protumor	-	Not evaluated	[50]
Prostate	Tumor tissue	Antitumor	Protumor	High nuclear HO-1 expression correlates with poorer overall survival	[68]
Breast	Tumor tissue	Antitumor	Protumor	Positive nuclear HO-1 expression correlates with higher histological grade	[9]
Head and neck	Tumor tissue	Protumor	Protumor	Positive nuclear HO-1 expression correlates with higher histological grade	[49]
Colon	Tumor tissue	Antitumor	Protumor ***	Not evaluated	[32]
Gliomas	Tumor tissue	Pro tumor	-	Not found(Insufficient number of samples?)	[48]
Multiple myeloma	Tumor tissue *	Antitumor	Protumor	Not applied	[58]
Chronic myeloid leukemia	Tumor tissue *	Antitumor	Protumor	Not applied	[59]

* tumor cell lines; ** acetyl-HO-1; *** observed in an animal model of CRC; SCC = squamous cell carcinoma; NSCLC = non-small cell lung carcinoma.

In breast cancer, our group recently demonstrated both cytoplasmic and nuclear HO-1 expression in human tumor biopsies, as well as in several in vitro and in vivo experimental models [9]. Heme Oxygenase-1 overexpression reduced tumor burden, induced cell cycle arrest and apoptosis, and decreased cell migration and invasion, impairing metastatic dissemination, which reflects an antitumor role. Moreover, HO-1 expression showed an association with longer overall survival time. On the contrary, although nuclear HO-1 expression failed to show an association with survival, a correlation with a higher histological grade has been found. These results suggest that nuclear HO-1 may have a role in more aggressive tumors and such role may be related to the protein itself instead of to HO-1 by-products, because nuclear HO-1 form showed a lack of enzymatic activity [9]. Interestingly, the expression of the proteases that cleave the C-terminal of HO-1, cathepsin B [77], calpain-2 [78] and SPP [79], have been associated with a poorer overall survival in breast cancer patients. However, whether any of these enzymes are implicated in HO-1 truncation in this type of tumor cells remains unknown.

In human Head and Neck Squamous Cell Carcinoma (HNSCC) tissue samples, cytoplasmic and nuclear expression of HO-1 was also observed [49]. In this tumor type, nuclear HO-1 expression was higher in tumor tissues compared to normal tissues, and HO-1 expression correlated with higher histological grades. Interestingly, an increase in nuclear expression of HO-1 was found in a carcinogenic murine model of squamous cell carcinoma where cytoplasmic HO-1 was expressed in pre-neoplastic lesions and nuclear HO-1 was expressed in tumor tissues [49]. These results suggest that nuclear HO-1 is associated with tumor progression and may play a protumor role. In accordance, ongoing studies from our group show that pharmacological treatment with hemin and genetic overexpression of HO-1 in HNSCC cells induces cell viability and cell cycle progression (unpublished results). Interestingly, it has been demonstrated that cathepsin B [80] and calpain-1 [81], both enzymes able to cleave HO-1, are overexpressed in this type of tumor and such expression has been identified as an independent unfavorable prognostic factor. However, the role of some of these cysteine proteases or SPP in HO-1 truncation in HNSCC cells remains to be demonstrated.

In CRC, HO-1 overexpression was demonstrated to reduce cell proliferation, increase cell cycle arrest and apoptosis, and reduce cell migration. Interestingly, such cell behavior depended on the presence of wild type p53, as p53 knock-out HCT116 and p53-mutated HT29 colorectal cancer cell lines failed to demonstrate these effects [32]. Furthermore, analyses of HO-1 expression on human biopsies have shown that positive HO-1 expression was higher in tumor cells than in non-adjacent tissues, and such expression correlated with a wild type K-ras status, normal carcinoembryonic antigen (CEA) levels and a longer overall survival time. Interestingly, nuclear HO-1 expression was found in tumor tissues but was absent in non-malignant adjacent tissues in human biopsies. Moreover, by using a chemically induced CRC model, it has been demonstrated that nuclear HO-1 expression increased throughout tumor progression from non-malignant, polyps, adenocarcinoma and signet-ring cell carcinoma tissues [32]. Therefore, and similar to head and neck, prostate and breast cancer, the nuclear compartmentalization of HO-1 in CRC, seems to associate with malignant behavior. In accordance with these results, Yin et al. reported a significant increase in cytoplasmic HO-1 expression in human colorectal tumor samples compared to normal colorectal tissue, and its correlation with well-differentiated tumors. Interestingly, the authors also found nuclear HO-1 expression in poorly/moderately differentiated tumors, which supports a protumor role of nuclear HO-1 [82]. In addition, HO-1 cleavage and dislocation from the sER by SPP were demonstrated in HCT116 cells [15] and overexpression of cathepsin B [83,84,85] and calpain-1 [86] associated with a reduced overall survival time.

In human glioma tissue samples, some malignant astrocytes have been shown to express cytoplasmic and nuclear HO-1. Moreover, an association of the increase in HO-1 expression with tumor aggressiveness and a significant HO-1 expression in astrocytoma and oligodendroglioma subtypes compared to normal brain tissue were found. However, although in astrocytoma positive HO-1 expression correlated with a shorter survival time, nuclear HO-1 expression did not associate with poor prognosis [48], although the number of samples analyzed was small.

Nuclear HO-1 was also reported in some hematological malignances such as Chronic Myeloid Leukemia (CML) and Multiple Myeloma (MM). In CML, the Abl kinase inhibitor imatinib induced apoptosis that was reversed when HO-1 was overexpressed, showing that HO-1 has cytoprotective effects in this type of cancer. Nevertheless, inhibition of the nuclear translocation of HO-1, not only reestablished imatinib effect on cell death but also increased it. Interestingly, HO-1 inhibition in imatinib-resistant CML cells induced apoptosis, which suggests that nuclear HO-1 is contributing to chemoresistance [59]. Similarly, in MM, the proteasomal inhibitor bortezomib was shown to induce HO-1 expression mediated by ER stress and increase its nuclear translocation. However, inhibition of HO-1 did not affect bortezomib effect on cell survival while inhibition of the nuclear translocation of HO-1 contributed to bortezomib cytotoxicity. These results show that HO-1 compartmentalization interferes with bortezomib treatment, which led to chemoresistance. Indeed, these authors demonstrated that a bortezomib-resistant cell line exhibited a higher expression of nuclear HO-1 compared to a bortezomib-sensitive cell line. In addition, the inhibition of nuclear HO-1 reduced the effect of bortezomib on chromosomal instability suggesting a role for nuclear HO-1 in DNA repair [58]. The inhibition of nuclear translocation by E64d inhibitor in both CML and MM suggests that a cysteine protease is involved in HO-1 compartmentalization. However, what kind of protease, whether calpain-1/2 or cathepsin B, is involved has not been studied in detail yet.

One of the types of cancer where nuclear HO-1 has been more thoroughly studied is lung cancer. Using a wide range of human non-small cell lung carcinoma tissues, Degese et al. [50] demonstrated that HO-1 is expressed in the cytoplasm and the nucleus of tumor cells being mainly expressed in the former compartment. Interestingly, in lung epithelial non-malignant tissues, HO-1 was expressed in the nuclear compartment, although the expression was very low. A similar observation about nuclear HO-1 staining pattern in lung cancer was also reported by other authors [87]. Heme Oxygenase-1 total protein expression was also associated with advanced tumor stages, T status and with lymph node metastasis but an association with patient survival has not been found. Nonetheless, these findings suggest that HO-1 may play a protumor role in lung cancer [50]. Later, Hsu et al. also reported nuclear HO-1 expression in human lung biopsies [88]. Interestingly, these authors demonstrated that HO-1 interacted with SPP, which cleaves HO-1 at its transmembrane segment. Indeed, SPP was highly expressed in lung cancer cells, was associated with a poorer overall survival [79], and correlated with HO-1 nuclear localization in the same lung cancer tissues [88]. As a consequence of the intramembranous cleavage of HO-1 by SPP, a t-HO-1 form was generated that underwent nuclear translocation [88]. Other potential enzymes able to cleave HO-1 as cathepsin B [89,90] and calpain-2 [91] were shown to be overexpressed in lung cancer, but their participation in HO-1 truncation remains to be demonstrated. Once inside the nucleus, t-HO-1 was able to induce cell proliferation, migration and invasion, independently of its enzymatic activity. Furthermore, these same authors shed some light on the molecular mechanisms underlying the nuclear HO-1 role. They found that lysine residues of t-HO-1 were acetylated by p300/CBP and that they could be deacetylated by classical HDACs. They also demonstrated that acetylated t-HO-1 bound to the JunD subunit of the AP-1 transcription factor. This posttranslational modification of nuclear HO-1 was shown to be required to induce cell proliferation, migration and invasion, and metastasis. Moreover, acetylated nuclear HO-1 was found in human lung adenocarcinoma biopsies but failed to be expressed in the normal counterparts [76]. In this regard, the authors postulated that in this type of tumor, the evaluation of acetylated HO-1 in human tumor biopsies has a potential clinical relevance as biomarker although they were not able to find a correlation between acetylated HO-1 expression and tumor progression. The authors concluded that the lack of correlation might be due to the small sample size they used and suggested that this study should be performed with a large number of samples before reaching a reliable conclusion.

## 5. Concluding Remarks and Future Perspectives

**Figure 2:**
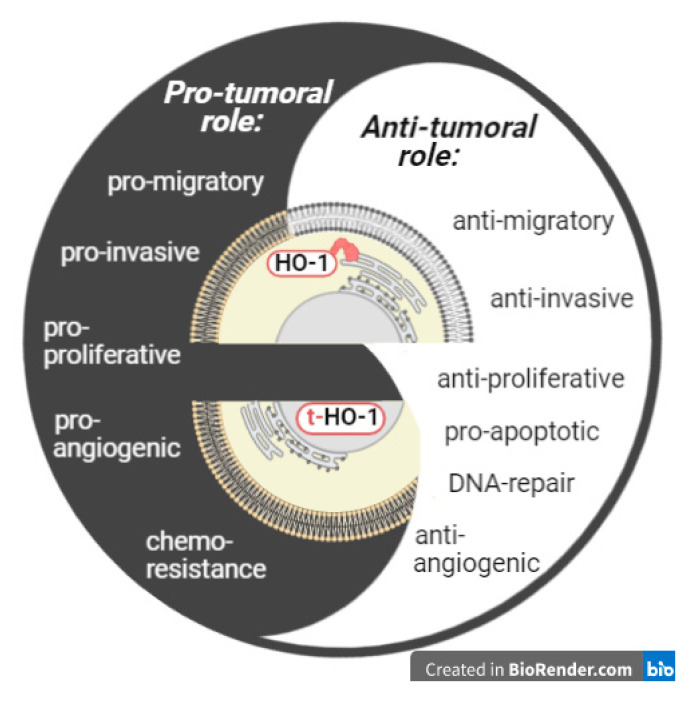
Subcellular compartmentalization of HO-1 conditions its role in malignancies. Full-length HO-1 resides in the sER, is enzymatically active and may play an antitumor or protumor role. Truncated HO-1 resides in the nucleus, lacks enzymatic activity and may play a protumor role.

Abundant evidence has shown opposite roles (protumor or antitumor) of HO-1 in cancer progression, although the underlying basis for this dual behavior remains mostly elusive. Cytoplasmic-nuclear shuttling of HO-1 has been demonstrated in several malignant as well as non-malignant conditions and may explain, at least in part, the dual role of HO-1 in cancer. Indeed, nuclear HO-1 has been associated with a protumor role in the majority of tumor types studied so far (Table 1). This protumor behavior of nuclear HO-1 may be due to the induction of some cellular processes such as cell proliferation, migration, invasion, metastasis, and chemoresistance, among others (Figure 2).

**Figure 3:**
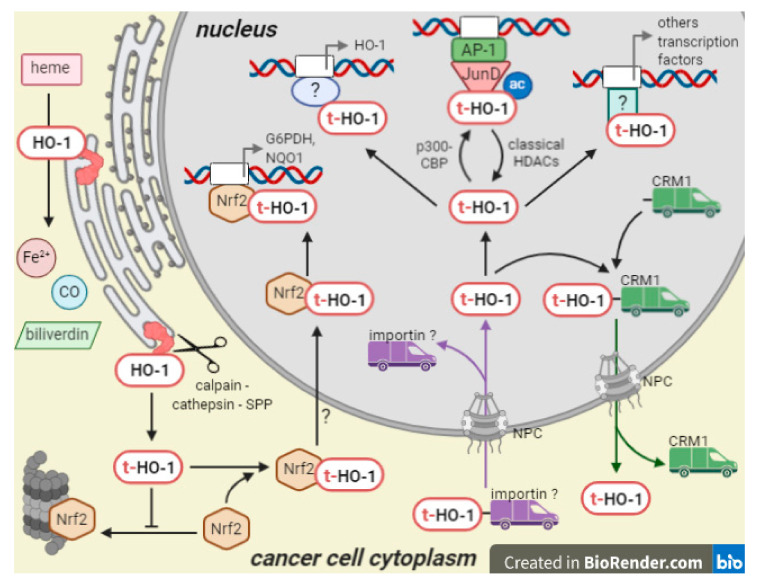
Mechanism of action of nuclear HO-1. Enzymatically active HO-1 is proteolitically cleaved from the sER and the generated t-HO-1 form migrates to the nucleus. Nuclear import of t-HO-1 is probably carried out by interaction with an importin, whereas nuclear export is carried out by interaction with CRM-1. Inside the nucleus, t-HO-1 may be acetylated by p300/CBP, binds to transcription factor as Nrf2 and AP-1 and activate them in order to transcribe genes related with antioxidant defenses and cell proliferation, among others.

Related to the mechanisms underlying HO-1 nuclear translocation (Figure 3), and although some aspects remain unexplored, studies on non-malignant and tumor cells showed that the proteolytic cleavage of the C-terminal of HO-1 from the sER by SPP or some other cysteine proteases, generating a truncated HO-1 form with reduced enzymatic activity, is essential for its translocation into the nucleus. Nonetheless, it remains to be investigated whether there is any pathological stimulus in a tumoral environment that can induce the proteolytic cleavage besides hypoxia. In addition, it is important to note that, to date, though the involvement of the cysteine proteases has been demonstrated by pharmacological inhibition, a more specific genetic knockdown has not been performed yet. Furthermore, to date, there is limited evidence regarding how t-HO-1 enters into and is exported from the nuclear compartment. Once into the nucleus, t-HO-1 is able to modulate its own gene promoter enhancing HO-1 expression. Moreover, t-HO-1 is acetylated by p300/CBP and this posttranslational modification is key to t-HO-1 binding to the JunD subunit and activation of the AP-1 transcription factor, which would contribute to cell proliferation and invasion. It has also been demonstrated that t-HO-1 interacts with Nrf2, and then such complex migrates into the nucleus and modulates transcriptional activity of antioxidant genes. However, it remains unknown whether a posttranslational modification of t-HO-1 is a conserved mechanism that conditions its action as a co-transcriptional regulator or impairs its binding to CRM-1, thus avoiding nuclear export and stabilizing its nuclear location (as observed for example for FOXO proteins [92]), thus potentiating its protumor effects.

From a translational perspective, understanding the molecular mechanisms of nuclear HO-1 might lead to the development of a targeted cancer therapy that prevents cytoplasmic to nuclear translocation of HO-1. Taking also into account that in several tumors nuclear HO-1 correlates with higher histological grades, this strategy may be useful to retard tumor progression and prolong the patient survival time. It would also be interesting to evaluate the nuclear HO-1 expression in tumor biopsies, further discriminating acetylated t-HO-1, in order to determine its usefulness as a biomarker to select those patients that may develop chemoresistance or may have a worse prognosis.
